# Molecular Evolution of the Porcine Type I Interferon Family: Subtype-Specific Expression and Antiviral Activity

**DOI:** 10.1371/journal.pone.0112378

**Published:** 2014-11-05

**Authors:** Yongming Sang, Joseph Bergkamp, Frank Blecha

**Affiliations:** Department of Anatomy and Physiology, College of Veterinary Medicine, Kansas State University, Manhattan, Kansas, United States of America; CSIRO, Australia

## Abstract

Type I interferons (IFNs), key antiviral cytokines, evolve to adapt with ever-changing viral threats during vertebrate speciation. Due to novel pathogenic pressure associated with *Suidae* speciation and domestication, porcine IFNs evolutionarily engender both molecular and functional diversification, which have not been well addressed in pigs, an important livestock species and animal model for biomedical sciences. Annotation of current swine genome assembly Sscrofa10.2 reveals 57 functional genes and 16 pseudogenes of type I IFNs. Subfamilies of multiple IFNA, IFNW and porcine-specific IFND genes are separated into four clusters with ∼60 kb intervals within the IFNB/IFNE bordered region in SSC1, and each cluster contains mingled subtypes of IFNA, IFNW and IFND. Further curation of the 57 functional IFN genes indicates that they include 18 potential artifactual duplicates. We performed phylogenetic construction as well as analyses of gene duplication/conversion and natural selection and showed that porcine type I IFN genes have been undergoing active diversification through both gene duplication and conversion. Extensive analyses of the non-coding sequences proximal to all IFN coding regions identified several genomic repetitive elements significantly associated with different IFN subtypes. Family-wide studies further revealed their molecular diversity with respect to differential expression and restrictive activity on the resurgence of a porcine endogenous retrovirus. Based on predicted 3-D structures of representative animal IFNs and inferred activity, we categorized the general functional propensity underlying the structure-activity relationship. Evidence indicates gene expansion of porcine type I IFNs. Genomic repetitive elements that associated with IFN subtypes may serve as molecular signatures of respective IFN subtypes and genomic mechanisms to mediate IFN gene evolution and expression. In summary, the porcine type I IFN profile has been phylogenetically defined family-wide and linked to diverse expression and antiviral activity, which is important information for further biological studies across the porcine type I IFN family.

## Introduction

Interferons (IFNs) are key cytokines that regulate immune responses against viral infection in vertebrates [1]–[5]. In mammals, interferons (IFNs) are diversified into three major types (i.e., type I, II and III) based on chromosomal locations, gene structures and sequence similarity as well as cognate receptors in target cells to discern IFN signaling [1], [2]. For example, the single member of human type II IFN, IFN-γ, has a gene containing four exons located in Chromosome 12; and the genes of three human type III IFNs (IFN-λ1 to IFN-λ3) contain five or six exons clustered in Chromosome 19 [1], [2]. In contrast, type I IFNs have evolved through a subtype expansion resulting in at least nine subclasses, including IFN-α, IFN-β, IFN-ε, IFN-κ and IFN-ω commonly found in most mammalian species as well as more species-specific branches IFN-δ (pigs and horses), IFN-ζ (mice), IFN-τ (cattle) and IFN-αω (pigs, horses and cattle) [1]–[8]. Moreover, subclasses including IFN-ω, IFN-δ, IFN-ζ, IFN-τ and particularly IFN-α have further diversified into multiple subtypes, such as humans having 13 functional IFN-α subtypes. Compared with the single or few gene loci of type II or III IFNs that flank 5–50 kb on a chromosome, the gene cluster of type I IFNs may comprise up to 50 single-exon (or intronless) IFN genes in each species and span 0.4–1 Mb on one or two chromosomes [1]–[8].

Mammalian type I IFNs probably emerged during tetrapod evolution from fish [9]–[11]. In zebrafish, several IFN progenitors named IFNphi have been identified [11]. These fish IFNs were inferred to be progenitors of type III IFNs due to their five-exon (or four-intron) gene structure; however, protein sequence analysis indicated that these fish IFNphi proteins contain signature motifs (such as four conserved cysteine residues and C-terminal CAWE motif) more like type I IFNs. Therefore, whether these fish IFNs serve as common ancestors of both type I and type III IFNs or as progenitors closer to either type is unclear [10], [11]. A recent study of IFNs in the African clawed frog indicated that type I and III IFNs were already diverged in amphibians, showing five genes of frog IFN-λ and five intron-containing IFN progenitors of type I IFNs located separately in different genomic scaffolds [9]. Genes of avian type I IFNs clearly are intronless, as characterized in about 10 chicken IFN-α and one IFN-β (originally designated as chicken IFN-1 and IFN-2, respectively) [12]. The intronless type I IFNs in amniotes appear to have arisen from a retroposition event that assumedly replaced the original type I IFN locus with intron-spliced RNA, thus favoring subsequent gene duplication and family expansion adaptable to rapidly evolving viruses and multifunctional divergence [9]–[12].

Type I IFNs are multifunctional despite their central role in antiviral responses [1]–[5], [13]–[16]. For example, although both human IFN-α and IFN-β constitute a primary antiviral mechanism in many cell types, the former is more potent in antiviral activity, and the latter is more active in anti-proliferation [1]–[5]. In this regard, IFN-ε was recently shown to be constitutively expressed by epithelial cells in the female reproductive tract, dynamically regulated by maternal hormones and unique in protecting the reproductive tract from viral and bacterial infection [17]. IFN-κ is predominately expressed and stimulated upon viral infection in keratinocytes, where it induces antiviral responses through unique action on cutaneous immune cells [18]. The other subtypes, including IFN-δ, IFN-τ, IFN-ω and IFN-ζ, have evolved specific niches in antiviral protection or other biological functions, particularly in some species [2], [3], [19]. Expanded IFN-ω genes appear in cattle, pigs, horses, and cats, where they display multiple functional genes compared with only one in humans; nearly a dozen feline IFN-ω variants have been associated with protection against parvovirus infection [2], [5], [6], [19], [20]. IFN-ζ, the subclass so far only found in mice, may act as an antiviral sentinel in bone marrow or the spleen due to its high antiviral and low lymphomyelo-suppressive activity [5]. IFN-δ and IFN-τ, two novel subclasses that arose recently in swine and ruminant species, respectively, are primarily released by the embryos prior to implantation and trigger gestation in the maternal uterine endometrium [1], [2], [5], [19].

Of porcine type I IFNs, IFN-α and IFN-β have been studied mostly for their response to and activity during viral infections [2], [8], [21]. Several subtypes of IFN-α and IFN-δ subclasses were identified through screening cDNA or genomic DNA libraries in some pioneering work, which inferred swine-specific divergence of type I IFN genes [3], [8], [19]. Overall identification of porcine IFN-α, IFN-δ and IFN-ω gene composition were facilitated by the swine genome project and clearly indicated active gene diversification in these porcine IFN subtypes [7], [8], [21]. We have previously annotated porcine type I IFNs family-wide using draft genome sequences, showing that the porcine type I IFN family has at least 39 functional genes, including 17 IFNA, 11 IFND and 7 IFNW as well as a single gene of IFNB, IFNE, IFNK and IFNAW (IFNA, IFNB, IFND, IFNE, IFNK, IFNW and IFNAW: gene symbols for IFN-α, IFN-β, IFN-δ, IFN-ε, IFN-κ, IFN-ω and IFN-αω, respectively) [21]. Notably, swine have conserved subclasses of IFN-β, IFN-ε and IFN-κ and dramatically diversified subclasses of IFN-α, IFN-δ and IFN-ω with rapidly expanded gene members, particularly species-specific expansion of IFN-δ [7], [8], [21]. Mammalian IFN-κ gene is generally separated distantly from the main cluster of IFN locus on the same chromosome; moreover, the porcine IFN-κ gene was positioned on chromosome (SSC) 10 instead of co-located on SSC1 with other subtypes [7], [8], [21]. IFN-αω, previously ascribed to the IFN-α subclass, was found to be a new subclass (found only in pigs, horses, and ruminants) based on its distinct phylogenic relationship and gene location relative to other subclasses [3], [21]. These findings imply an active, evolving process in the porcine type I IFN family, which deserves further study to refine its molecular diversity within the newly released swine genome assembly (Sscorfa10.2) [7], [8]. Here, we show as many as 57 functional genes and 16 pseudogenes of type I IFNs distributed on Sscorfa10.2; interestingly, these 57 functional IFN genes include 18 duplicates, each identical to one of the previously identified 39 IFN genes. Molecular evolutionary analyses indicate that porcine type I IFN genes have been undergoing active diversification due to both gene duplication and conversion. Obvious gene expansion under purifying selection and genomic association with some repetitive elements were significant in IFN-α, IFN-ω and IFN-δ subclasses, which contain multiple functional genes. Further family-wide expression and activity assays attest to functional divergence across the expanding porcine type I IFN family, which evokes target-optimization of IFN activity pertaining to molecular and functional diversity. In summary, data presented here not only update the molecular composition of porcine type I IFNs based on the newly assembled swine genome and our previous study [7], [8], [21], they also advance our understanding of the porcine type I IFN family through extensive analyses of molecular evolution, expression and restrictive activity against porcine endogenous retrovirus.

## Results and Discussion

### Gene composition of porcine Type I IFNs, potential duplicates and pseudogenes

**Figure 1:**
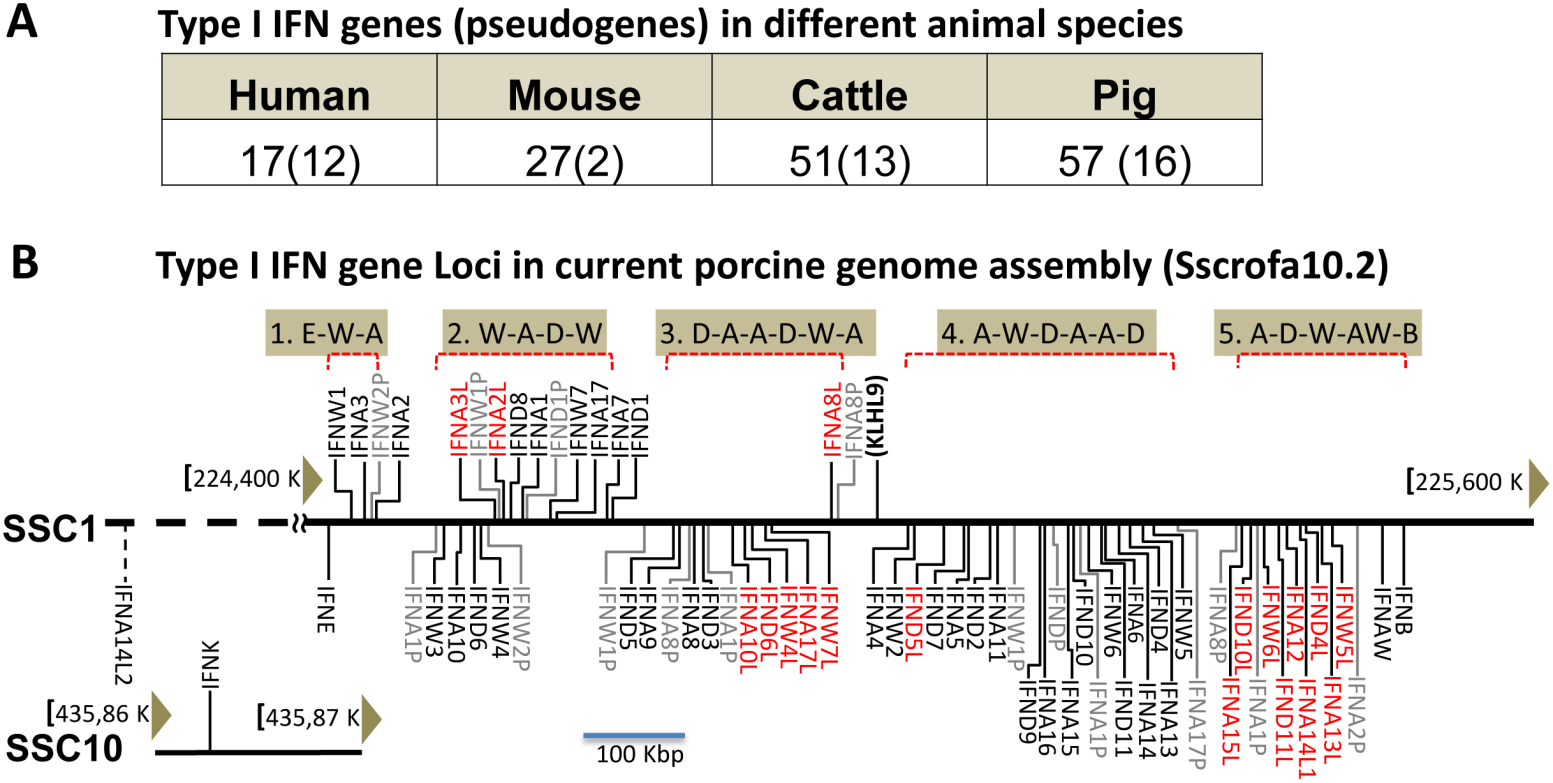
Gene composition of type I IFN loci. (A) Type I IFN functional gene (pseudogene) numbers in different animal species imply gene expansion of porcine type I IFN genes. (B) Distribution of porcine type I IFN genes in the current porcine genome assembly. There are 18 duplicates (labeled in red), each identical to one of the previously identified IFN genes; therefore, there are 39 parsimoniously non-redundant porcine type I IFN genes plus 18 duplicates resulting either from very recent gene duplication events or artifactual assembly. Gene symbol legend: black, functional genes; grey, pseudogenes; red, duplications to the indicated ones (for examples, IFNA1L has 99–100% sequence identity to IFNA1; dashed line, unassembled piece. KLHL9, kelch-like protein 9-like gene in the middle of type I IFN gene locus.

Our objective was to determine the molecular diversity of porcine type I IFN genes, and to correlate this to their expression and function to porcine development and immunity. Extensive annotation of the current swine genome assembly (Sscrofa10.2) identified a total of 57 type I IFN functional genes plus 16 pseudogenes, clearly indicating gene expansion comparable to that in cattle but much larger than the gene composition of type I IFNs in humans and mice (Fig. 1A) [7], [8], [21]. Each of these porcine IFN functional genes contains an intact open reading frame (ORF) potentially encoding a protein precursor of 153–208 amino acids (AA), which is characterized to have a conserved IFab domain, a signature domain of type I IFNs. In contrast, all pseudogenes have a fragmental exon (or ORF) or premature in-frame stop codons, despite showing high sequence similarity to some IFN functional genes. As shown in Figure 1B, all porcine type I IFN gene loci, except IFNK, are clustered in an SSC1 region spanning about 1 Mb. Similar to gene loci of type I IFNs characterized in other animal species, porcine type I IFN loci are bordered by two genes of more primitive origin, IFNB and IFNE, as well as marked by the gene of kelch-like protein 9 (KLHL9) in the middle of the IFN gene cluster. To either side of the KLHL9 gene locus, 36 or 37 IFN genes (including pseudogenes) are grouped into five clusters separated by four blank interval regions at ∼60 Kb/each. In contrast to human and mouse type I IFN gene loci, where IFN clusters are predominant with few subtypes of IFNA (or IfnA and IfnZ in mice) genes, porcine type I IFN gene clusters are mingled by genes of several subtypes, mostly IFNA, IFND and IFNW. From left to right, for example, cluster 1 contains genes of IFNE, IFNW and IFNA, clusters 2 to 4 have multiple genes of IFND, IFNA and IFNW, and cluster 5 contains five subtypes of IFNA, IFND, IFNW, IFNAW and IFNB. Three to four pseudogenes appear randomly within each cluster, but only one is found in cluster 1. Hypothetically, the initiation and expansion of multiple genes of porcine IFN-α, IFN-δ and IFN-ω subclasses within the IFNB/IFNE margins could undergo a simultaneous rather than a sequential process of diversification. In other words, the progenitors of porcine IFNA, IFND and IFNW genes could have generated at a close temporal scale, then duplicated and diversified parallel in each sub-cluster. The expansion of porcine type I IFN genes resembles what occurs in the bovine genome except for species-specific differences in IFN subtypes; for instance, bovine type I IFN loci contain as many as 24 IFNW, 3 IFNT and 6 IFNB genes. As in most animal species, the swine genome contains only one IFNB gene. The emergent IFNAW gene, which encodes the IFN-αω subtype (termed IFN-µ in horses [6]) and is phylogentically more related to IFN-α and IFN-ω, has, thus far, been found only in pigs, horses and some ruminants [2], [21], [22]. The phylogenic specificity of IFN-αω has been postulated in analysis of bovine type I IFNs and demonstrated in pigs and horses [6], [21], which infers some natural selection pressure experienced commonly during evolution (and possibly domestication) in these species. Another feature of the porcine type I IFN gene loci is that IFNK is located in SSC10 instead of distantly located in the same chromosome as in other species. The unexpected finding of a porcine IFNK locus on a different chromosome than other type I IFNs was confirmed by annotation of the current swine genome assembly and probably resulted from chromosome relocation during porcine evolution. This is implied by a radiation hybrid map of SSC10 defined a syntenic region to human chromosome 9 (HSA9) at 27–99 Mb, which contains the human IFNK gene locus [21], [23]. However, relocation of porcine IFNK to another chromosome may not provide a spatial reason for favoring porcine type I IFN gene expansion because bovine type I IFN gene loci experienced a similar expansion despite IFNK appearing linearly in the same chromosome [2], [7], [21], [22].

Further examination of the 57 porcine type I IFNs functional genes revealed 18 duplicates, which contain ORFs encoding protein precursors identical to one of the 39 previously defined IFNs. These duplicates belong to the subclasses of IFN-α, IFN-δ and IFN-ω with multiple genes. To examine whether these duplicates are assembly artifacts or authentic porcine IFN paralogs, we extracted the genomic sequence adjoining each ORF for approximately 4 Kb, which flanks 2 Kb 5′-upstream and 1 Kb 3′-downstream from the ORF and tentatively includes major gene elements of promoter and untranslated regions. Despite the high identity of ORF (84.8–97.1%, Table 1) and protein sequences (74.0–95.0%), the average similarity of IFN genes of the 4 Kb sequences within a subclass is approximately 70%, indicating a higher variance of gene elements than the ORFs. In contrast, all duplicates share >98% identity throughout the entire gene sequence (Table 1). Based on this observation and the similarity threshold, we tentatively ascribe these 18 duplicates of functional genes to assembling artifacts; otherwise, these gene duplications might have occurred very recently in domestic pigs and are worth verifying in the genomes of wild pigs [24]. All 18 duplicates and pseudogenes are akin to the multi-gene subclasses of IFN-α, IFN-δ and IFN-ω, indicating higher diversification of these subclasses compared with the single-gene subclasses of IFN-β, IFN-ε and IFN-κ (Fig. 1B). Even excluding these 18 duplicates, pigs still have at least 39 type I IFNs non-redundant at the protein level. This result is consistent with our previous annotation using draft genomic sequences [21] and clearly implies gene expansion of porcine type I IFN genes compared with that in humans and mice (Fig. 1A). The IFN-αω, and the multiple subtypes of IFN-δ and IFN-ω, consist of species-specific characteristics in the molecular composition of porcine type I IFNs, and thus deserve more study to determine their functional propensity. Collectively, analyses of current swine genome assembly have identified uncommonly high molecular diversity of type I IFN family than other species, but comparable with cattle [7], [8].

**Table 1:** Similarity (ClustalW Score) to the closest paralogues.

	Sscrofa 10.2 Coordinate	Gene* %	ORF %	Protein %
All IFNA-average		72.5	97.1	95.0
IFNA3L	224582359 (−) 224582904	98 (A3)	100 (A3)	100 (A3)
IFNA2L	224593324 (−) 224593869	99 (A2)	99 (A2)	100(A2)
IFNA17L	224642712 (−) 224643257	90 (A17)	99 (A17)	100 (A17)
IFNA10L	224816424 (+) 224816969	99 (A10)	100 (A10)	100 (A10)
IFNA8L	224912405 (−) 224913232	99 (A8)	100 (A8)	100 (A8)
IFNA15L	225313077 (+) 225313643	100 (A15)	100 (A15)	100 (A15)
IFNA12	225355681 (+) 225356317	100 (A6)	100 (A6)	100 (A6)
IFNA14L	225368677 (+) 225369246	100 (A14)	100 (A14)	100 (A14)
IFNA13L	225389979 (+) 225390548	100 (A13)	100 (A13)	100 (A13)
All IFND-average		69.8	84.8	74.0
IFND6L	224827505 (+) 224828059	100(D6)	100(D6)	100(D6)
IFND5L	225020781 (+) 225021335	100(D5)	100(D5)	100(D5)
IFND10L	225321632 (+) 225322147	100(D10)	100(D10)	100(D10)
IFND11L	225351081 (+) 225351635	100(D11)	100(D11)	100(D11)
IFND4L	225376601 (+) 225377116	99(D4)	100(D4)	100(D4)
All IFNW-average		73.4	91.3	82.8
IFNW4L	225225692 (+) 225226264	99(W4)	100(W4)	100(W4)
IFNW7L	224853285 (+) 224853857	100 (W7)	100 (W7)	100 (W7)
IFNW5L2	225400323 (+) 225400895	100(W5)	100(W5)	100(W5)
IFNW6L	225334442 (+) 225335014	100(W6)	100(W6)	100(W6)

* Putative genes were generated from genomic regions flanking the ORF plus 2 kb 5′-upstream and 1 kb 3′-downstream sequences adjacent to the ORF. Protein sequences were predicted from the ORFs. The near identical sequences at gene level may indicate that the paired paralogues are duplications occurred very recently or some assembling artifacts.

### Phylogenic analysis

**Figure 2:**
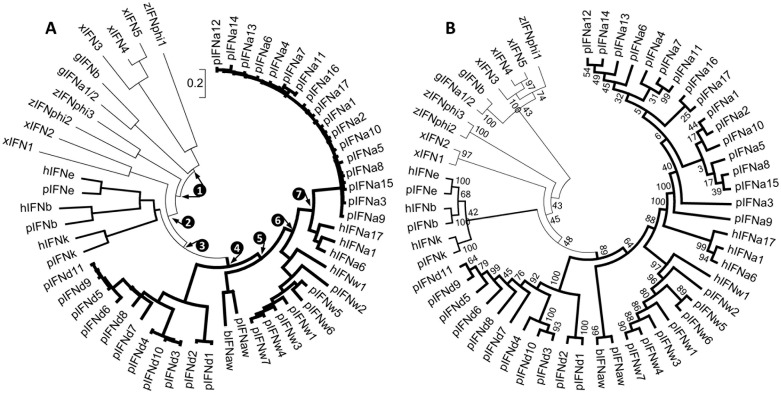
Phylogenic analysis of porcine type I IFNs in cohort with orthologs from other species. Evolutionary analyses were conducted in MEGA5 [27] to show relative branch length (A) and tree topology with bootstrap values (B). Internal nodes corresponding to the hypothetical common ancestors of mammalian (primarily porcine) type I IFNs evolved from lower vertebrate IFN-like molecules are illustrated using circled numbers around the center. The bootstrap consensus tree inferred from 1000 replicates is taken to represent the evolutionary history of the taxa analyzed. The evolutionary history was inferred using the Neighbor-Joining method. Bootstrap values are indicated along each branch. The evolutionary distances were computed using the Poisson correction method and are in the units of the number of amino acid substitutions per site. Tree branch and taxa legend: Tree branches of solid lines with increased width illustrate the evolution of type I IFNs from IFN ancestors in zebrafish (z), Xenopus (x) and Gallus (g) to diverse mammalian subtypes exemplified with porcine (p) type I IFNs and some typical orthologs in human (h) or bovine (b). IFN taxa used, IFNa, IFNaw, IFNb, IFNd, IFNe, IFNk and IFNw correspond to IFN-α (IFNA), IFN-αω (IFNAW), IFN-β (IFNB), IFN-δ (IFND), IFN-ε (IFNE), IFN-κ (IFNK) and IFN-ω(IFNW), respectively, in classic nomenclature for IFN protein (gene) precursors.

Phylogenic analysis of type I IFNs in pigs and other species implies both a paralogous relationship within the porcine subclasses and cross-species orthologous speciation [7]–[12]. IFN-like molecules recently have been identified in lower vertebrate species of fish, frogs and birds [9]–[12]. To retrace their ancestral relationship with porcine type I IFNs, three zebrafish IFNs (zIFNphi1–3) and five frog (*Xenopus tropicalis)* IFNs (xIFN1–5) were included in our phylogenic analysis. As shown in Figure 2, these IFN-like molecules of lower vertebrates indeed form monophyletic groups discretely linked to chicken (*Gallus gallus*) IFNs (gIFNs) and selected mammalian type I IFNs, primarily porcine types. Notably, zIFNphi1 forms a clade with xIFN3–5 and chicken gIFNs, whereas the other two zebrafish IFNs, zIFNphi2 and zIFNphi3, have a closer relationship with amphibian xIFN1 and xIFN2 and, thus, are more ancient than mammalian type I IFNs. Considering that genes of both zebrafish and frog IFN precursors contain several introns, but genes of chicken and mammalian type I IFNs are intronless, the retroposition events leading to loss of introns in IFN genes could have occurred independently in birds or mammals because their type I IFNs evolved from different prototypes [10]. Bordering or distantly located from other IFN clusters in mammalian genomes, genes of mammalian IFN-ε, IFN-β and IFN-κ have been postulated to be more primitive than other subclasses with multiple subtypes, such as IFN-α in all mammalian species as well as IFN-δ and IFN-ω in pigs [10], [21], [22]. Why mammalian IFN-ε, IFN-β and IFN-κ loci remain latent whereas other subtypes such as IFN-α genes undergo active gene diversification [9]–[12], [21], [22] remains elusive. Parallel with the prototypes of IFN-ε, IFN-β and IFN-κ, the common ancestor (indicated by the circled 4 in Fig. 2A) of porcine IFN-α, IFN-δ and IFN-ω subtypes seem to evolve rapidly in subtype formation and isoform multiplication. Clearly reflected from the phylogenic tree in Fig. 2, the porcine IFN-δ subclass branched earlier into a different clade than IFN-α and IFN-ω subclasses that have subdivided more recently along with the emergence of the intermediate subtype of IFN-αω. The furcation of some IFN-δ (IFN-δ5, -δ6, -δ9 and -δ11) and, in particular, the expansion of an entire porcine IFN-α subclass, might have happened very recently, as shown by the collapsed branches (Fig. 2A) as well as non-significant bootstrap values (<50) to support the branch topology (Fig. 2B). In sequence similarity, porcine type I IFNs of one subclass have sequence identities higher than 80% nucleic acid or 60% amino acid, and sequence similarity between subclasses is lower than 70% nucleic acid or 50% amino acid. In addition, orthologs from different mammalian species (such as IFN-ε, IFN-β and IFN-κ from humans and pigs) were grouped in the same terminal clades, indicating an even closer phylogenic relationship between them than between different subclasses in the same species. IFN-αω, which was previously found in pigs and cattle, and recently detected in horses, has intermediate phylogeny but only ∼50% amino acid similarity to either IFN-α and IFN-ω. IFN-δ comprises a specific subclass only detected in pigs and horse so far, which has an atypical cysteine-motif (according to the locations of the second and third Cys residues in protein sequences) compared with other type I IFN subclasses and shows its closest phylogenic relationship to bovine IFN-τ and murine IFN-ζ subtypes, with near 60% amino acid identity in general (data not shown) [21]. Overall, our phylogenic analysis implies functional domain conservation underlying active subtypic diversification of type I IFNs in vertebrate evolution, and in particular, shows evolutionary relationship of porcine type I IFNs in the manner of within- and cross-species.

### Gene conversion and natural selection

In addition to gene duplication proposed as a major mechanism for IFN diversification, gene conversion, which refers to fragmental replacement between homologous sequences (either allelic or ectopic), also might be involved in evolution of the type I IFN gene family. Using the ORF-centered 4 Kb-long gene pieces (tentatively including 5′-promoter and 3′-untranslated contexts) extracted from the NCBI genome database (http://www.ncbi.nlm.nih.gov/genome/), we analyzed gene conversion among porcine type I IFN sequences. Gene conversion was analyzed with GENECONV software using the default setting to compute both the Sim P-value (estimated based on permutation) and the Bonferroni-corrected (BC) KA (BLAST-like) P-value [25]. Table 2 lists pairs of porcine type I IFN gene sequences that had one or both P-values less than 0.05, thus indicating significant potential for gene conversion within the comparative gene domains, including tentative regulatory sequences at 5′- and 3′-termini as well as the coding ORFs. Specifically, detected gene conversion between IFN-δ genes all occurred in the ORF and 3′-terminal regions, whereas genes of IFN-α and IFN-ω contain converted segments mostly within the terminal regulatory sequences. Typically, the gene conversion within ORF coding regions might entail more similar proteins, and those in the terminal regulatory regions might render more expressional synergy. Accordingly, IFN genes with conversions generally composed a monophyletic group in the phylogenic tree (Fig. 2). In addition, to confirm the accuracy of the above gene conversion detected using GENECONV, we analyzed the same sequence alignment files with multiple inbuilt algorithms in the RDP3 package [26]. As shown in Table S2, many more recombination events (including most significant ones detected by the GENECONV discussed above) were detected by the multiple algorithms; however, some hits could be either actual recombinants or misidentification [26].

**Table 2:** Gene conversion analysis for porcine type I IFN genes.

				Aligned*	
Group	Sequence names	Sim P-value	BC KA P-value	Begin	End	Length (Domain)	Number of Polymorphisms	Total of Differences
**SsIFNAs**	IFNA7;IFNA11	0.0010	0.02168	3820	4904	1085 (orf+3′)	194	13
	IFNA1;IFNA2	0.0014	0.02834	4060	4743	684 (orf+3′)	57	44
	IFNA13;IFNA15	0.0155	0.17097	3850	3996	147 (5′)	43	50
	IFNA17;IFNA15	0.0379	0.36441	3876	4008	133 (5′)	37	54
								
**SsIFNDs**	IFND4;IFND10	0	0	4586	4953	368 (3′)	206	216
	IFND4:IFND3	0	0.00011	3547	3785	239 (orf)	95	254
	IFND4;IFND3	0	0.00011	4555	4739	185 (3′)	95	254
	IFND4;IFND10	0.0001	0.00134	3542	3785	244 (orf)	98	216
	IFND9;IFND6	0.0002	0.00214	4460	4611	152 (3‘)	56	356
	IFND5;IFND6	0.0004	0.00435	3978	4092	115 (3′)	55	348
	IFND5;IFND6	0.0011	0.00781	3583	3712	130 (orf)	53	348
	IFND4;IFND10	0.0011	0.00884	3815	4030	216 (orf)	87	216
	IFND4;IFND3	0.0012	0.00997	3815	4000	186 (orf)	73	254
	IFND11;IFND8	0.0014	0.01243	4072	4325	254 (3′)	41	426
	IFND4;IFND10	0.0047	0.02928	4389	4584	196 (3′)	80	216
	IFND5;IFND6	0.0068	0.0451	4480	4611	132 (3′)	47	348
								
**SsIFNWs**	IFNW3;IFNW4	0	0	1697	2028	332 (5′)	168	138
	IFNW3;IFNW4	0	0.00018	4083	4498	416 (3′)	139	138
	IFNW6;IFNW3	0.0001	0.00052	552	1050	499 (5′)	82	217
	IFNW7;IFNW4	0.0094	0.0415	2205	2509	305 (5′)	29	426

Gene conversion was analyzed with GENECONV [25] the default setting. Both the Sim P-value based on permutation and Bonferroni-corrected (BC) KA (BLAST-like) P-values were used to estimate gene conversion among compared homologues. The significant pairs with either or both P-values <0.05 were listed. The gene-converted fragments in 5′-, 3′-terminal, or/and open reading frame (ORF) regions were aligned. The polymorphic sites and mismatched sites between gene-converted fragments were listed under “number of polymorphisms” and “total of differences”.

**Figure 3:**
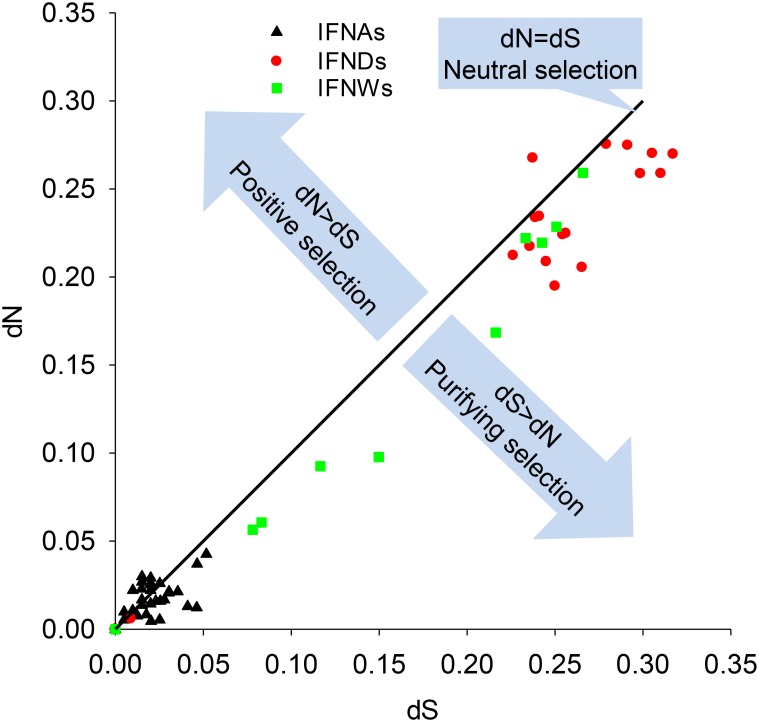
Selection pressure on the coding regions of porcine type I IFNs. Each gene pair in the IFN gene subfamilies containing multiple-function genes (i.e., IFNA, IFNW and IFND subfamilies) were pairwise analyzed using a dN/dS calculation tool (http://services.cbu.uib.no/tools/kaks) for calculation of synonymous (dS) or non-synonoymous (dN) changes in the coding regions, and the dS and dN values were plotted against one another. The center diagonal line represents neutral selection rate where dS = dN. Gene pairs undergoing positive selection (dN>dS) appear above the diagonal line and gene pairs undergoing purifying selection (dS>dN) appear below the diagonal. Significant estimation (p<0.05) of positive and purifying selections were calculated using MEGA5 [27] and listed in bold in **Table 3** in comparison with the selection pressure on all members of the IFN subfamilies.

Natural selection places pressure on biological molecular evolution to co-opt the species-specific gene composition of type I IFNs [10]. Comparing the rate of non-synonymous nucleotide changes (dN) to synonymous changes (dS) implies the type of selection operating on the members of multiple gene families. Therefore, a dN:dS = 1 defines a neutral selection indicating all nucleotides in the sequences randomly mutated equally. A dN:dS ratio greater than 1 indicates a positive selection implying rapid changes in amino acids to accommodate an emerging novel function; whereas, a ratio less than 1 implies a purifying selection that results in no amino acid change [7], [25], [27]. We computed dN:dS values of porcine type I IFNs using two different algorithms, as shown in Figure 3 and Table 3 [25], [27]. Purifying selection, which related to pairwise comparisons of multi-gene subfamilies of porcine IFN-α, IFN-ω, and particularly IFN-δ, was significantly (p<0.05) detected. In addition, overall computation among the ORFs of all genes within IFN-α, IFN-ω and IFN-δ subfamilies manifested a general tendency of purification selection rather than positive selection, showing *p* values of 1.0 for positive selection and 0.283–0.011 for purifying selection, as well as much lower dN than dS values (Table 3, the three rows at the bottom of the blocks of positive and purifying selection, respectively). Pairwise comparison revealed that most IFN-δ genes and three pairs of IFN-α or IFN-ω genes display the consequences of significant purification selection (Table 3). As plotted in Figure 3, purifying selection was predominant within the genes of IFN-ω and IFN-δ subfamilies, whereas more positive selection pressure was detected among the genes of IFN-α subfamily; although only pairwise comparison of IFNA2 and IFNA3 was significant (Table 3). Similar to IFNs of humans and mice, most members of the porcine IFN-α subfamily and IFN-β are more ubiquitously expressed in many tissues and cell types, but IFN-α members generally exert higher antiviral activity, whereas IFN-β has higher anti-proliferative or immunomodulatory activity [1], [4]–[6], [28]. Among other porcine subtypes, their distinct function remains elusive; nonetheless, some members of porcine IFN-δ and IFN-ω have been associated with reproductive regulation [19], and porcine IFN-ε and IFN-κ likely conserves the tissue-specific virostatic activity as shown with their murine orthologs in the female reproductive tract and cutaneous layers, respectively [17], [18]. Therefore, the pathogenic pressure accompanying viral infections during swine evolution might contribute to the tendency of positive selection to emerge in the porcine IFN-α subfamily (Figure 3); in contrast, genes of other subtypes more specific to immune or development regulation could be eventually fixed through purifying selection [4]–[6], [19], [28]. Interestingly, pairwise comparison also revealed significant positive selection between IFND9 and IFND11 (Table 3), which deserve further study for their association with potential tissue- or pathogen-specific functions. In addition to the natural selection analyses based on intra-species paralogs, more extensive analyses based on cross-population variants will infer a better demonstration of selection pressures on IFN genes. In this context, Manry et al. (2011) [29] found that some human IFN-α subtypes have evolved under strong purifying selection; in contrast, selective constraints have been relaxed for other type I IFNs such as IFN-α10 and IFN-ε. It is likely that similar results would occur if the selection pressure on porcine IFN subtypes is analyzed among polymorphic variants across swine breeds worldwide.

**Table 3:** Positive and purifying selection in porcine IFNAs, IFNDs and IFNWs.

Pairwise analysis group	p-value	dN-dS
**Positive selection**		
IFNA2; IFNA3	**0.016**	2.159
IFND9; IFND11	**0.010**	2.361
Overall IFNDs	1.000	-2.323
Overall IFNAs	1.000	-0.577
Overall IFNWs	1.000	-1.622
**Purifying selection**		
IFNA2; IFNA17	**0.045**	-1.707
IFNA8; IFNA17	**0.040**	-1.769
IFNA15; IFNA17	**0.030**	-1.906
IFND1; IFND7	**0.008**	-2.431
IFND2; IFND7	**0.008**	-2.431
IFND3; IFND5	**0.029**	-1.912
IFND3; IFND6	**0.010**	-2.355
IFND3; IFND7	**0.000**	-3.770
IFND3; IFND9	**0.048**	-1.677
IFND3; IFND11	**0.043**	-1.732
IFND4; IFND6	**0.009**	-2.384
IFND4; IFND5	**0.025**	-1.974
IFND4; IFND7	**0.000**	-3.913
IFND4; IFND9	**0.043**	-1.736
IFND4; IFND11	**0.038**	-1.790
IFND5; IFND6	**0.017**	-2.142
IFND6; IFND7	**0.008**	-2.456
IFND6; IFND10	**0.019**	-2.097
IFND7; IFND10	**0.000**	-3.620
IFNW3; IFNW6	**0.044**	-1.719
IFNW4; IFNW6	**0.029**	-1.921
IFNW6; IFNW7	**0.020**	-2.069
Overall IFNDs	**0.011**	-2.323
Overall IFNAs	0.283	-0.577
Overall IFNWs	0.054	-1.622

### Association with repetitive elements within sub-loci

Repetitive elements compose over half of typical mammalian genomic sequences and are located mostly in non-coding regions with unknown functions [7], [8]. Repetitive elements recently have been implicated in gene duplication by creating syntonic regions predisposed to homologous recombination [29], [30]. Some types of repetitive elements were potentially involved in interchromosomal assembly of enhanceosomes to amplify human IFN-β gene expression and targeted by NF-κB transcription factors to regulate antimicrobial immune responses [29], [30]. Further information from the Encyclopedia of DNA Elements (ENCODE) project has determined that 80% of the human genome (mostly non-coding sequences) is now associated with at least one biochemical function, and much of this functional non-coding DNA acts to regulate the expression of coding genes [32]. Thus, repetitive elements in proximal regions of IFN loci may comprise phylogenic marks and a regulatory mechanism for IFN gene expression [7], [22], [31]. Estimated by the common algorithm of RepeatMasker [33], the 1.1 Mbp region of the porcine type I IFN locus contains nearly 30% interspersed repeats, less than the approximately 40% in the whole swine genome or murine type I IFN locus [7]. The most abundant types of repetitive elements are the long interspersed nucleotide elements (LINEs), accounting for 14.80%; next are the short interspersed nucleotide elements (SINEs), which constitute 7.73%; and endoretroviral elements (ERV) and other DNA elements account for nearly 4% and 3%, respectively (Table 4).

**Table 4:** Repetitive elements within the porcine type I IFN locus of ∼1.1 Mbp region in Chromosome (SSC) 1.

	Number ofelements *	Length occupied (bp)	Percentage ofsequence
**SINEs:**	425	82096	7.73%
Alu/B1	0	0	0.00%
MIRs	64	8139	0.77%
**LINEs:**	258	157158	14.80%
LINE1	198	142930	13.46%
LINE2	57	13991	1.32%
L3/CR1	3	237	0.02%
RTE	0	0	0.00%
**LTR elements:**	131	42385	3.99%
ERVL	11	1743	0.16%
ERVL-MaLRs	62	20622	1.94%
ERV_ClassI	57	19795	1.86%
ERV_ClassII	0	0	0.00%
**DNA elements:**	89	32810	3.09%
hAT-Charlie	61	28850	2.72%
TcMar-Tigger	5	1321	0.12%
**Unclassified:**	0	0	0.00%
**Total interspersed repeats:**		314449	29.60%
**Small RNA:**	3	299	0.03%

The repetitive elements were detected with RepeatMasker version 3.3.0.

using default mode [31]. *most repeats fragmented by insertions or deletions were counted as one element.

We further analyzed the repetitive elements specifically associated with the subtypes of porcine type I IFN genes (Table S1 and Table 5). Corresponding repetitive elements were remarkably associated with each subtype of IFN genes, such as inclusion of one or several SINE or DNA-transposon-type repetitive elements in front of most IFNA, IFND and IFNW genes. Medium reiteration frequency interspersed repeats (MERs), for example, are a group of DNA transposons and retrotransposons at appropriately 0.5–1 kb, and mammalian-wide interspersed repeats (MIRs) belong to a group of SINEs that are several hundred base-pairs long. As such, one to three MER106B were shown to be associated with all IFNA genes (except IFNA17) within 3 kb upstream of the coding region of each IFN gene. Another MERs member, MER96B, was detected in about half of the IFND genes; the other half of the IFND genes and all IFNW genes are associated with MIR/MIRb/MIRc of the SINE class. In contrast to the MER106B elements adjoining the proximal promoter regions (∼500 bp prior to the ORF) of IFNA genes, most MER96B and MIR elements occur distantly in front of IFND or IFNW genes at 4–8 kb from the coding regions (Table S1). No typical elements of MER or MIR families were detected in front of the single-gene subtypes of IFNE and IFNB genes; however, an endogenous retrovirus long terminal repeat 54 (LTR54) and a Tigger15a transposable element were located within 1 kb upstream of IFNE and IFNB coding regions, respectively. The intermediate subtype, IFNAW, like IFNW genes, contains a MIR instead of MER106B in its tentative promoter region (Table S1).

**Table 5:** Association of repetitive elements with porcine type I IFN genes in SSC1.

IFN gene	Associated repetitive element*	Phylogenic relevance** (Overall topological score)
IFNAs	MER106B	63.40%
IFNDs	MER96B/MIRc/MIRb	75.50%
IFNWs	MIR/MIRb/MIRc	82.30%
IFNAW	MIR	n.a.
IFNB	MIR	n.a
IFNE	LTR54	n.a
		
IFNAPs	MER96B/MER106B/L1MCB_5	<50%
IFNDPs	MER96B	<50%
IFNWPs	L1MCB_5/MIR/L1ME_ORF2	<50%

*The association of repetitive elements was examined with the Censor program [31]. **The phylogenies of Newick strings of both IFN genes and associated repetitive elements were generated using the MEGA[26], and topological comparison between the Newick trees was performed with Compare2Trees at (http://www.mas.ncl.ac.uk/~ntmwn/compare2trees).

In addition to subtype-specific association with IFN genes spatially, we also showed that these repetitive elements have a co-evolutionary relationship with their allied IFN genes. By comparing the topologies of phylogenic trees generated from IFN genes and their allied repetitive elements using the same phylogenic algorithm [27], [34], we showed that MER106B elements shared 63.40% topological similarity with allied IFNA genes, and even higher topological similarities of 75.50% and 82.30% were detected for IFND genes and IFNW genes, respectively, with their associated repetitive elements (column of Phylogenic relevance, Table 5, and Figure S1). In contrast, the topological comparison between the phylogenic trees of IFN pseudogenes and their associated repetitive elements (either conserved or non-conserved with functional genes) revealed no notable relevance, indicating the reduction of spatial or phylogenic relevance between these IFN pseudogenes with potential regulatory repetitive elements (Table 5). Therefore, because repetitive elements help mediate genomic reorganization and gene duplication, the spatial and phylogenic association of typical repetitive elements with different IFN subtypes may indicate their role in IFN duplication origins and evolutionary correlation, which may be particularly meaningful for studying duplication mechanisms of the multi-gene IFN subtypes [7], [22]. The reduced association between IFN genes with their typical repetitive elements or re-organized association with other types of repetitive elements in multiple IFN pseudogenes indicates that this kind of intra-gene association may be critical for keeping an IFN gene functional or de-functioning during the purifying selection process. In addition, because of these subtype-specific repetitive elements mostly located within (MER106B in IFNA genes, LTR45 in IFNE, and Tigger5 in IFNB gene) or next to (MER96B or MIRs in IFND and IFNW genes) putative promoter regions of IFN genes, they may frequently constitute *cis*-elements to regulate IFN gene expression [30]. The single-gene subtypes of IFNB and IFNE, bordered at two ends of the porcine IFN locus in SSC1, have different repetitive elements adjoining their promoter regions. This also indicates that re-organization of MER and MIR elements with the prototypes of multi-gene IFN subtypes might facilitate the duplication process to form multi-gene IFN subclasses [10], [21], [22], [30].

### Subtype- and isoform-specific differential expression

**Figure 4:**
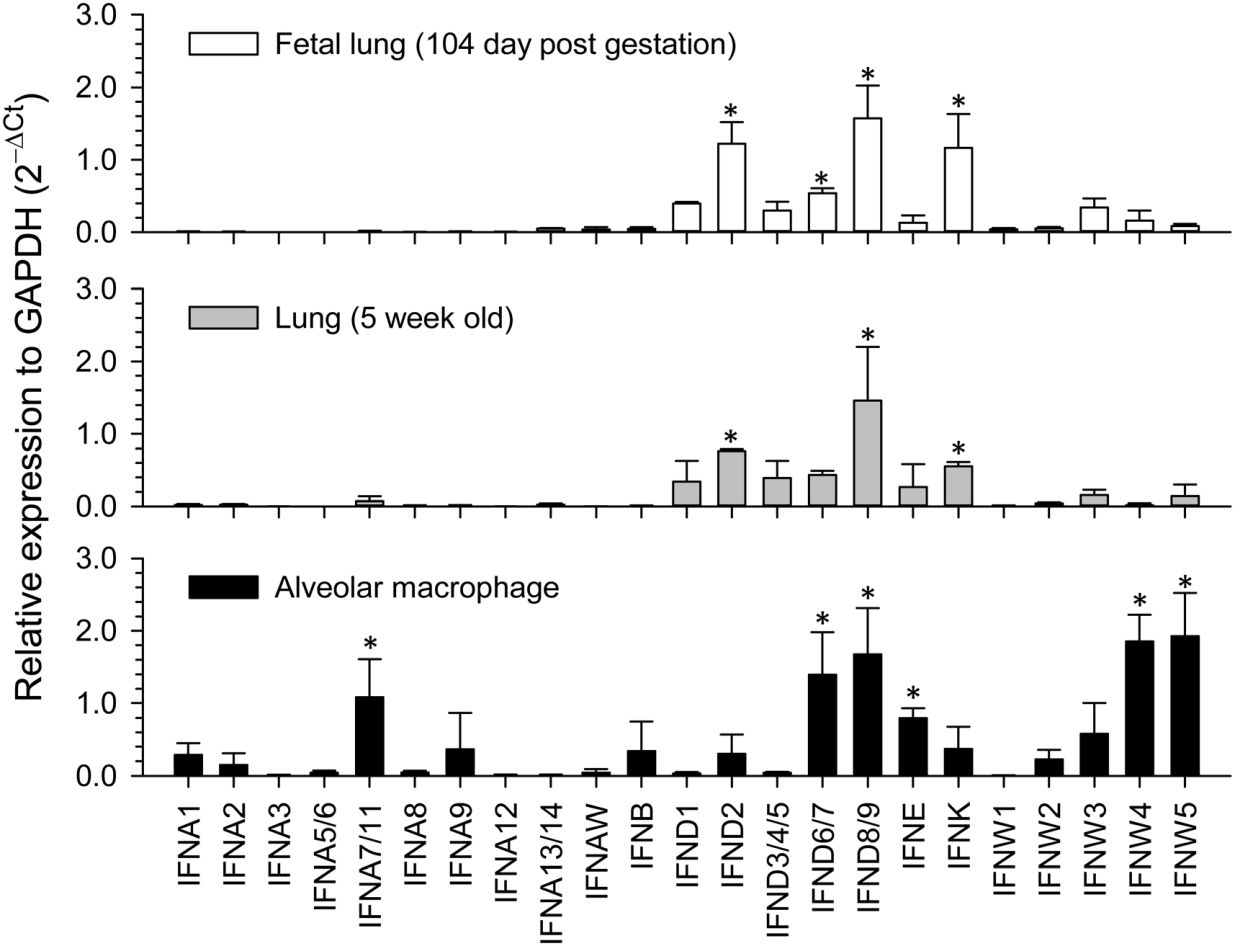
Family-wide expression analysis of porcine type I IFN genes in porcine lungs and alveolar macrophages. Gene expression was analyzed using a SYBR Green-based real-time RT-PCR assay. Total RNA (100 ng) was used in each 20 µl of PCR reaction. Ct values of the genes were normalized against Ct values of a housekeeping gene (GAPDH) amplified from the same RNA samples to obtain 2^−ΔCt^, reflecting relative expression of each IFN gene. Data are means ± SE; n = 3 replicates, *p<0.05 to average expression level of all IFN genes. Primers for RT-PCR detection are as previously described [21].

Using a quantitative RT-PCR array validated in a previous report [21], we examined family-wide differential expression of porcine type I IFNs in porcine lungs and alveolar macrophages, major leukocytes in the lungs (Figure 4). In healthy lungs from developed fetuses at the later phase of gestation or from 5-wk-old pigs, all IFNA genes had only slight expression, with IFNA7/11 being notably higher. In contrast, IFNE, IFNK, multiple genes of IFNW and particularly IFND genes were constitutively expressed and much higher than IFNA or IFNB genes. In differentiated alveolar macrophages, the major phagocyte and IFN-α producer after respiratory virus infections [35], we observed higher expression of IFNA and IFNW genes than in the lungs. This expression pattern is similar to our family-wide activity assay against both porcine reproduction and respiratory virus (PRRSV) and vesicular stomatitis virus (VSV) [21], in which most IFN-α and some IFN-ω subtypes exerted higher antiviral activity and showed differential expression response to PRRSV infection (data not shown). In addition, porcine IFN-δ, IFN-ε and IFN-κ, as well as IFN-ω subtypes, which generally showed constitutive expression but less antiviral activity than IFN-β and particularly IFN-α, may reflect more activity for immunomodulation, development and metabolic regulation [5], [19], [28]. It is imperative to establishing high-throughput procedures based on “omics” technology to family-wide profile IFN expression in different tissues, at different developmental stages and for comparisons between infected and non-infected states [21], [36].

### Subtype- and isoform-specific antiviral activity

**Figure 5:**
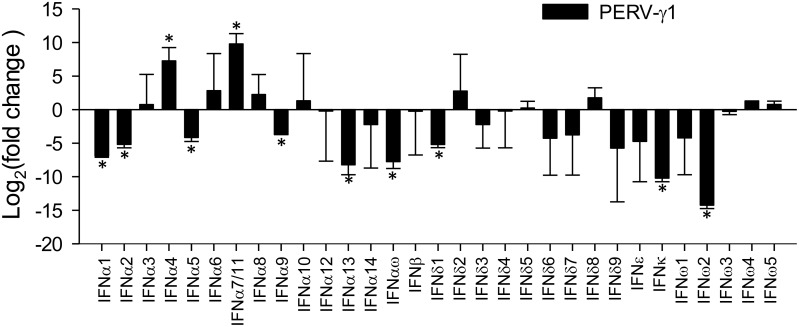
Restriction of PERV-γ1 replication in porcine PK-15 cells. Cells were treated with different IFN peptides at 20 ng/ml for 60 h, and RNA was extracted to determine the resurgence of PERV RNA using a FastLane Cell SYBR Green Kit (Qiagen, Valencia, CA) using published primers specifically for the PERV-γ1 subtype (including PERV-A, -B and –C) [40]. Real-time RT-PCR data were processed and plotted to show fold changes relative to the control cells. Data are means ± SE; n = 4 independent assays, *p<0.05 relative to the mock treatment controls.

Endogenous retroviral elements (ERV) are remnants of ancestral retroviral integration into the genome of germ-line cells constituting 4–10% of genome sequences in different animal species [7], [33], [37]–[39]. The expression of ERVs is closely checked by cellular epigenetic factors at the DNA level and vigorously restricted by immune mechanisms such as those mediated by IFNs [37], [38]. Accordingly, inappropriate expression or resurgence of ERVs has been shown to drive chronic inflammation in some cancers, autoimmune disorders, and viral infections [37], [38]. In pigs, the resurgence of Class I γ-type retrovirus has been a major biosafety concern during xenotransplantation to human patients [39]. To compare the restrictive activity of porcine type I IFNs on ERV resurgence, we examined the expression of porcine endogenous retrovirus (PERV)-γ1 (including PERV-A, PERV-B and infectious PERV-C) in a PK-15 cell line after 60 h treatment with IFN peptides [7], [21], [40]. As shown in Figure 5, most porcine type I IFNs generally suppressed PERV-γ1 expression 2–15 logarithm units of fold changes at the tested dose (20 ng/ml). Surprisingly, the IFN-α subtypes, which generally showed much higher antiviral activity against PRRSV and VSV in our previous report [21], were less active than the other IFN subtypes in suppressing PERV-γ1 expression. Some very effective IFN-α members against PRRSV and VSV, such as IFN-α3, IFN-α4, IFN-α6, IFN-α8 and IFN-α10, showed little actual suppression and even an increase in PERV-γ1 expression in the PK-15 cells compared with the mock controls (Figure 5). Most porcine type I IFNs effectively suppressed the ancient ERV resurgence, but some newly emerged IFN-α subtypes, which evolved to exert higher virostatic efficacy against contemporary porcine viruses (i.e., PPRSV and VSV), may lose activity to ERVs (Figure 5). If this is the case, the higher ERV-suppressive isoforms of IFN-α1, IFN-α2, IFN-α9 and IFN-α13 could represent prototypes of the porcine IFN-α subtype, which is difficult to determine based solely on phylogenic analysis (Figure 2). Therefore, corresponding to their phylogenic diversity and differential expression, porcine type I IFNs may further maximize their functional spectrum to confer subtype- and isoform-specific antiviral activity against different virus species [21], [39], [40]. Notably, we have previously conducted family-wide antiviral assays against porcine viruses such as PRRSV and VSV [21]. Collectively, the antiviral activity of porcine type I IFNs is not only subtype- and isoform-dependent, but also different for each virus.

### Evolutional view of potential structure-activity relationship of porcine Type I IFNs

**Figure 6:**
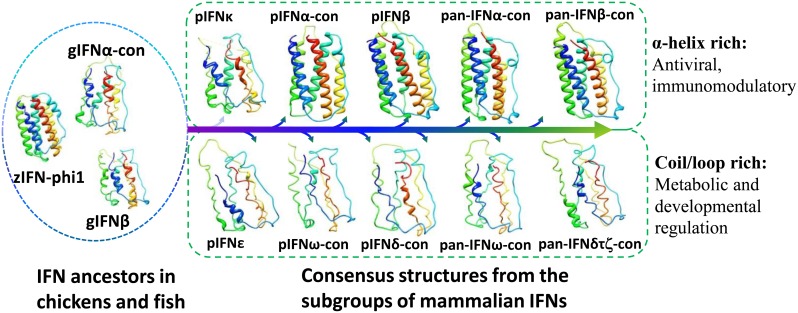
Structural association among lower vertebrate IFN ancestors and mammalian Type I IFN subtypes. Consensus sequences (IFN-con) were generated using multiple alignment programs of MUSCLE [44] and Jalview [43] and manually curated to represent most conservative residues among the aligned IFN peptides. The 3-D structures were modeled based on the structures of closely related homologs in structure databases using ESyPred3D [42]. Generally, all type I IFN peptides have a typical structure containing 5 α-helices; however, the high-antiviral subtypes such as most mammalian IFN-α and -β have higher α-helice density (upper panel above the arrow implying evolution) compared with IFN-ε, -ω and –δ/τ subtypes (bottom panel) that contain more coil/loop structures connected to small α-helices, which generally show lower antiviral but higher developmental regulation. Therefore, structural manipulation preferentially promotes antiviral functions. Species abbreviations: Dr, *Danio rerio;* Gg, *Gallus gallus;* Ss, *Sus scrofa;* Pan, multiple mammalian species.

To reveal the potential structure-activity relationship, we collected approximately 200 representative IFN sequences from lower vertebrates to main mammalian species deposited in the NCBI protein database (http://www.ncbi.nlm.nih.gov/protein/). The subtype consensus sequences (IFN-con) within or across species were generated and manually curated to represent most conservative residues among the aligned IFN peptides. The 3-D structures of individual or consensus IFN peptides were modeled based on the structures of closely related homologs in structure databases [41], [42]. As shown in Figure 6, all type I IFN peptides generally have a typical structure containing 5 α-helices, but the high-antiviral or -immunomodulatory subtypes such as most mammalian IFN-α and IFN-β have higher α-helices density (upper panel above the arrow) compared with IFN-ε, IFN-ω and IFN-δ/τ subtypes (bottom panel) that contain more coil/loop structures connected to small α-helices, which generally show lower antiviral but higher developmental regulation activity. In this context, we showed that the primitive IFN-phi1 in zebrafish has a higher density of α-helices, similar to mammalian IFN-α and -β structures; whereas, chicken IFN-α and IFN-β structures contain more coil/loop structures connected to small α-helices. Although it is difficult to distinctly determine the exact ancestors of each subtype of mammalian type I IFNs in lower vertebrates, this general analysis of the structural-activity relationship along the evolution stream suggests basic structural conservation in contrast to the ever-diversifying process driven by natural selection pressure and preferentially promotes structural manipulation for cross-species optimization of antiviral and immunomodulatory functions of type I IFNs [1], [4], .

## Conclusions

Emerging evidence indicates gene expansion of porcine type I IFNs, which might result from both gene duplication and conversion and active evolution through natural selection (particularly purifying selection) [8], [10], [11], [22]. Some genomic repetitive elements showed significant association with multi-gene IFN subtypes, which may serve as molecular signatures of respective IFN subtypes and genomic mechanisms to mediate IFN gene evolution and expression [21], [22], [30], [31]. Structurally, most mammalian IFN-α and IFN-β subtypes have a higher density of α-helices than IFN-ε, IFN-ω and IFN-δ/τ subtypes that contain more coil/loop structures connected to small α-helices, which generally show lower antiviral but higher developmental regulation activity [28]. In summary, the porcine type I IFN profile has been defined family-wide and compared phylogentically with orthologs from other species to discern the subtype-specific differentiation linking to expression and antiviral activity. This information will be important in conducting further biological studies across the expanded porcine type I IFN family.

## Methods

### Manual annotation and bioinformatic analyses of the porcine Type I IFN family

Annotation was done primarily with Otterlace/Zmap manual annotation software via web-based interaction with the Wellcome Trust Sanger Institute (WTSI) SingleSignOn system (affiliated with the Immune Response Annotation Group (IRAG) of the swine genome project) [7], [8]. The coordinated process of manual annotation, professional quality check, and sequence data analyses across mRNA, EST and Protein sequence databases as well as Ensembl predictions were systematically lineated to aid the annotation as described [8]. In addition, some porcine IFN entries were extracted from the NCBI gene database (http://www.ncbi.nlm.nih.gov/gene/) and further curated using BLASTP against the current swine genome assembly (Sscrofa10.2) [7], [8]. Conservation of the IFab domain coded by each ORF was evaluated against the Conserved Domain Database [41]. Sequence similarity and multiple sequence alignments were carried out using the Jalview and web-interactive MUSCLE program with default parameters [43]. For tree-building using Mega 5 [27], the neighbor-joining method was used to construct the phylogenetic tree by calculating the proportion of amino acid differences (p-distance), and the reliability of each branch was tested by 1,000 bootstrap replications. The evolutionary distances were computed using the Poisson correction method and are in the units of the number of amino acid substitutions per site. In addition, we generated consensus sequences (IFN-con) using multiple alignment programs of MUSCLE [44] and Jalview [43] and manually curated them to represent most conservative residues among the aligned IFN peptides. The 3-D structures were modeled based on the structures of closely related homologs in structure databases using ESyPred3D [41], [42].

### Duplication analysis

As previously defined, genes that yielded a one:many relationship during the orthology search were subjected to an additional round of similarity analysis. Artifactual duplication status was designated when genes possessed approximately 99% identity at the nucleotide level and were in a cluster of proximal genes tandemly duplicated at the same level of identity [8].

### Gene conversion and selective pressure analyses

Gene conversion was analyzed with GENECONV [25] at the default setting. The program identifies highly similar sequence subsets within aligned sequences and determines their statistical significance by computing a global P-value and a local P-value. In addition, it reports the length and location of these subsets predictably containing gene conversion. Those with P-values lower than a threshold (the default is <0.05) are considered to be indicative of gene conversion events. Because the global P-value is calculated based on consideration of all sequences in the alignments, as opposed to the pairwise P-value, which is based on consideration of only the pair of sequences of interest, global comparison is considered a more conservative method than pairwise comparison [25]. Supporting evidence of the polymorphic sites and mismatched sites between gene-converted fragments is reported under “number of polymorphisms” and “total of differences.” Because it is well-known that gene recombination detection is very program-dependent, we also examined gene recombination using multiple inbuilt algorithms (including RDP, BootScan, Maxchi, and SiScan) in the RDP3 program with the default setting as instructed [26].

Each gene pair in the IFN gene subfamilies containing multiple function genes (i.e., IFNA, IFNW and IFND subfamilies) were analyzed pairwise using a dN/dS calculation tool (http://services.cbu.uib.no/tools/kaks) for calculation of synonymous (dS) or non-synonoymous (dN) changes in the coding regions, and the dS and dN values were plotted against one another. Significant estimation (p<0.05) of positive and purifying selections were also calculated using MEGA5 [27].

### Identification of repetitive elements associated with porcine type I IFN sub-loci

About 1.1 Mbp region in Chromosome (SSC) 1 containing the porcine type I IFN locus was extracted from NCBI Reference Sequence NC_010443.4, and repetitive elements within were determined using the RepeatMasker program (version 3.3.0, http://www.repeatmasker.org/) in default mode. The IFN sub-locus association of repetitive elements was examined with the Censor program [33] using phylogenies of Newick strings of both IFN genes and associated repetitive elements generated with the MEGA [26]. A topological comparison between the Newick trees was performed with Compare2Trees at (http://www.mas.ncl.ac.uk/~ntmwn/compare2trees) [34].

### Differential Expression in pig lungs and macrophages

All recombinant DNA procedures and animal procedures were approved by the Kansas State University Biosafety and Institutional Animal Care and Use committees. Animal usage, PCR optimization, and real-time RT-PCR analysis were performed as described [21]. In brief, gene-specific or subtype-common primers were designed based on multiple alignments of related IFN sequences, and PCR conditions were optimized and validated using confirmed IFN plasmids to show specific amplification only with templates containing confirmed IFN clone(s). RNA was extracted from tissues and cells obtained from previous studies [21]. Real-time RT-PCR arrays in a 96-well microplate format (iCycler5.0, Bio-Rad, Hercules, CA) were performed with the validated primers [21]. Reactions were conducted with a SYBR Green RT-PCR system (Qiagen, Valencia, CA) with 150 ng of total RNA in a 25-µl reaction mixture. Specific optic detection was set at 78°C for 15 s after each amplification cycle of 95°C for 15 s, 56–59°C for 30 s and 72°C for 40 s. Critical threshold (Ct) values and melt curves were monitored and collected with iCycler 5.0 software, and final products after 40 PCR cycles were analyzed on agarose gels. Relative gene expression was first normalized against Ct values of the housekeeping gene (GAPDH) and compared with the expression levels of control samples [21].

### Differential activity in restriction of the resurgence of porcine endogenous retroviruses

We previously reported differential protective activities of porcine type I IFNs against PRRSV and VSV [21]. Here we demonstrate that porcine type I IFNs have different levels of activity in suppressing the resurgence of PERV RNA in PK-15 cells. In brief, cells were cultured in flat-bottom 96-well plates to 95% confluence, and 10 µl of each IFN peptide solution (20 ng in culture medium or medium only for controls) was added to a well containing 90 µl of fresh medium. At 60 h after incubation, cell RNA was extracted and the resurgence of PERV RNA was examined with a FastLane Cell SYBR Green Kit (Qiagen, Valencia, CA). In brief, RNA from cells of cultured wells was prepared using the FastLane lysing buffer to stabilize cellular RNA and eliminate genomic DNA, and FastLane lysates were used directly as templates in SYBR Green-based real-time one-step RT-PCR to detect the resurgence of PERV RNA using the published primers specifically for the PERV-γ1 subtype (including PERV-A, -B and –C) [33]. Real-time RT-PCR data were processed as above and plotted to show fold changes compared with control cells.

## Supporting Information

Figure S1 **Topological comparison between phylogenic trees generated using IFN genes (gene regions: ORF, open reading frame; 5′-UTR, 3 kb genomic pieces immediately upstream IFN ORFs) and their associated repetitive elements.** The phylogenies of Newick strings of both IFN genes and associated repetitive elements were generated using the MEGA [27], and topological comparison between the Newick trees was performed with Compare2Trees at (http://www.mas.ncl.ac.uk/~ntmwn/compare2trees). The overall topological scores (at the bottom of each comparison) between the IFN genes and allied REs were reported in Table 5.
(PDF)

Table S1 **Repetitive elements (RE) associated with the genes of porcine type I IFN subtypes.** The genomic sequence was extracted from chromosome 1 (SSC1) contig NC_010443.4 (http://www.ncbi.nlm.nih.gov/nuccore/NC_010443.4|:224375846–225698870). The association of repetitive elements (REs) was examined with the Censor program http://www.girinst.org/censor/index.php) to screen the 1.3 Mbp sequence on SSC1, which contains all porcine Type I IFN genes except the IFNK gene. The program reported the name, class, and length of the detected REs. The “similarity value (Sim)” reports the number of matches in the alignment normalized by the length of the alignment, whereas the “positive value (Pos)” reports the normalized number of alignment positions that produce positive scores in the alignment matrix. The location of either REs or IFN genes (ORFs) is related to the nt positions in the 1.3 Mbp region, and “distance to IFN ORF” was obtained to subtract the start position of a IFN ORF from the position of the closer end of the corresponding RE(s); the negative numbers indicate REs posited prior to 5′-end of the ORFs, and positive numbers for that after 3′-end of the ORFs. The IFN genes and corresponding REs are organized according to the order localized on SSC1, as shown in Figure 1.
(XLSX)

Table S2 **Gene recombination events deteted by the RDP3 package** **[43].** In brief, multiple sequence alignment files generated from the MUSCLE [42] programs for GENECONV assay were examined for gene recombination using multiple algorithms (including RDP, BootScan, Maxchi, and SiScan) built-in in a combinative RDP3 program with the default setting as instructed [26]. Results were converted from the RDP3 output files and, the definitions of abbreviations in the header row were listed at the bottom of the sheet.
(XLSX)
