# Supplementary material for: Molecular Evolution of the Porcine Type I Interferon Family: Subtype-Specific Expression and Antiviral Activity
Source: PLoS One. 2014 Nov 5;9(11):e112378. doi: 10.1371/journal.pone.0112378 (PMC4221479; doi:10.1371/journal.pone.0112378)
Supplement: Figure S1 — Topological comparison between phylogenic trees generated using IFN genes (gene regions: ORF, open reading frame; 5′-UTR, 3 kb genomic pieces immediately upstream IFN ORFs) and their associated repetitive elements. The phylogenies of Newick strings of both IFN genes and associated repetitive elements were generated using the MEGA [27], and topological comparison between the Newick trees was performed with Compare2Trees at (http://www.mas.ncl.ac.uk/~ntmwn/compare2trees). The overall topological scores (at the bottom of each comparison) between the IFN genes and allied REs were reported in Table 5. (PDF) [file pone.0112378.s001.pdf]

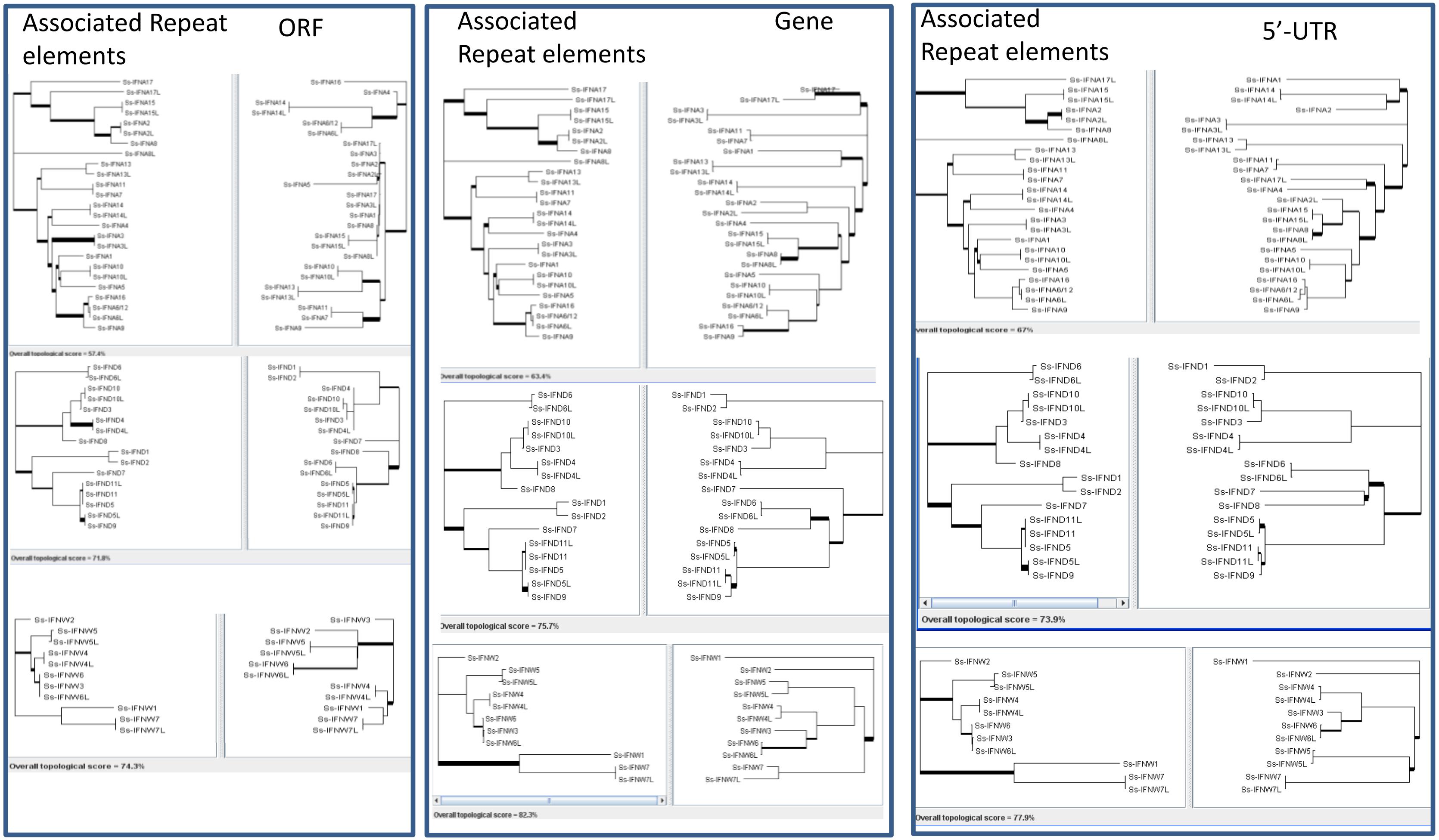

Figure S1: Topological comparison between phylogenic trees generated using IFN genes (gene regions: ORF, open reading frame; 5'-UTR, 3 kb genomic pieces immediately upstream IFN ORFs). The phylogenies of Newick strings of both IFN genes and associated repetitive elements were generated using the MEGA[4]; and topological comparison between the Newick trees was performed with Compare2Trees at (http://www.mas.ncl.ac.uk/~ntmwn/compare2trees). The overall topological scores (at the bottom of each comparison) between the IFN genes and allied REs was reported in Table 5.
